# Intuitive Judgments in Depression and the Role of Processing Fluency and Positive Valence: A Preregistered Replication Study

**DOI:** 10.32872/cpe.v2i4.2593

**Published:** 2020-12-23

**Authors:** Carina Remmers, Johannes Zimmermann, Sascha Topolinski, Christoph Richter, Thea Zander-Schellenberg, Matthias Weiler, Christine Knaevelsrud

**Affiliations:** a Division of Clinical Psychological Intervention, Department of Education and Psychology, Freie Universität Berlin, Berlin, Germany; b Department of Psychology, University of Kassel, Kassel, Germany; c Social and Economic Cognition Center, University of Köln, Köln, Germany; d Vivantes Klinikum Kaulsdorf, Berlin, Germany; e Division of Clinical Psychology and Epidemiology, Department of Psychology, University of Basel, Basel, Switzerland; Philipps-University of Marburg, Marburg, Germany

**Keywords:** depression, intuition, meaning detection, positive affect, positive valence, processing fluency, replication, semantic coherence judgments

## Abstract

**Background:**

Recent preliminary evidence indicates that depression is associated with impaired intuitive information processing. The current study aimed at replicating these findings and to move one step further by exploring whether factors known as triggering intuition (positivity, processing fluency) also affect intuition in patients with depression.

**Method:**

We pre-registered and tested five hypotheses using data from 35 patients with depression and 35 healthy controls who performed three versions of the Judgment of Semantic Coherence Task (JSCT, Bowers et al., 1990). This task operationalizes intuition as the inexplicable and sudden detection of semantic coherence.

**Results:**

Results revealed that depressed patients and healthy controls did not differ in their general intuitive performance (Hypothesis 1). We further found that fluency did not significantly affect depressed patients’ coherence judgments (H2a) and that the assumed effect of fluency on coherence judgments was not moderated by depression (H2b). Finally, we found that triads positive in valence were more likely to be judged as coherent as compared to negative word triads in the depressed sample (H3a), but this influence of positive (vs. negative) valence on coherence judgments did not significantly differ between the two groups (H3b).

**Conclusion:**

Overall the current study did not replicate findings from previous research regarding intuitive semantic coherence detection deficits in depression. However, our findings suggest that enhancing positivity in depressed patients may facilitate their ability to see meaning in their environment and to take intuitive decision.

## Abstract (highlights)

- The pre-registered replication study did not find intuition deficits in patients with depression.
- Processing fluency did not affect coherence judgments in depressed patients or healthy controls.
- Depressed patients and healthy controls use positive valence as cue for intuitive coherence judgments.
- Future studies should test whether enhancing positivity in depressed patients boosts their ability to find meaning (e.g., meaning in life).

People continuously make decisions and judgments without long consideration by relying on their gut feelings. Following one’s intuition does not only *feel* right (Thompson et al., 2011), but also leads to adaptive outcomes, especially in complex situations, during stress or when a person is experienced in the given environment (Kahneman & Klein, 2009). By integrating a multitude of factors such as implicit personal needs and goals (Baumann & Kuhl, 2002; Lieberman et al., 2004), intuitions enable people to make "smart" decisions (Gigerenzer, 2007), and to interact with other people (e.g., facilitating adaptive parent-child interaction; Parsons et al., 2017). Moreover, intuition is associated with central aspects of mental well-being, such as experiencing meaning in life (Heintzelman, Trent, & King, 2013; Hicks & King, 2007; Hicks et al., 2010; Schlegel et al., 2011).

Intuition relies on processes that are based on experience, run quickly and unconsciously, and allow many relevant aspects to be effortlessly integrated into a coherent whole (Kahneman, 2011). By this, intuition enables people to detect coherence and meaning. The Judgment of Semantic Coherence Task (JSCT; Bowers et al., 1990) operationalizes this core characteristic of intuition by asking people to discriminate word triads in terms of their semantic relatedness. Research using this task consistently shows that people can intuitively discriminate semantically related word triads (e.g., deep salt foam; common denominator: sea) from semantically unrelated word triads (e.g., dream ball book; no common denominator) (Bolte & Goschke, 2005; Bowers et al., 1990) without being able to explicitly name the basis for their judgment – they just know it without knowing why (Epstein, 2008).

It seems reasonable to assume that people are not always able to intuitively detect meaning and coherence. One important influencing factor seems to be the affective state of a person. Positive mood broadens the scope of attention, facilitates associative processing (Fredrickson & Losada, 2005; Harel, Tennyson, Fava, & Bar, 2016) and increases the preference for thematic processing that is needed for semantic coherence detection (e.g., Maldei, Baumann, & Koole, 2020). In line with this, people are more likely to rely on intuition (Zander-Schellenberg, Remmers, Zimmermann, Thommen, & Lieb, 2019) and are more accurate in discriminating meaning from meaninglessness when in a positive mood (Balas et al., 2012; Bolte et al., 2003). Negative mood states, in contrast, are associated with narrowing attentional focus and inhibiting associative processing (Sass et al., 2012). Along this line, negative mood and a tendency to brood have been shown to impair intuition (Baumann & Kuhl, 2002; Bolte et al., 2003; Remmers & Zander, 2018; Sweklej et al., 2015). Here, we assume that intuitive processing is impaired in depressed patients in particular.

Depression is characterized by negative mood and a brooding, rigid, style of thinking. This is opposed to an intuitively integrating and holistic style of processing (see Remmers & Michalak, 2016). While intuitive processing is accompanied by cognitive ease, feelings of rightness and the detection of meaning and coherent structures in the environment (see fluency-affect intuition model; Topolinski & Strack, 2009a), depressive thinking is doubtful – as a consequence, nothing feels easy and right anymore. Furthermore, depression is associated with experiencing less meaning in life and with lower abilities to construct coherent narratives of one’s life (Baerger & McAdams, 1999; Mascaro & Rosen, 2005). As finding meaning is mainly a product of intuitive processing (Hicks & King, 2007), and intentional, analytical search for meaning can impair the intuitive detection of meaning (Topolinski & Strack, 2008), it seems reasonable to assume that intuitive meaning detection is impaired in depression where a ruminative processing style is prevalent (Watkins & Teasdale, 2004). Recent research has indeed shown that patients with depression have deficits to intuitively detect semantic coherence as compared to healthy control participants (Remmers & Michalak, 2016; Remmers, Topolinski, Buxton, Dietrich, & Michalak, 2017; Remmers, Topolinski, Dietrich, & Michalak, 2015). The current study seeks to replicate these findings and moves one step further in exploring the underlying mechanisms of assumed intuition impairments in depression.

Apart from the influence of people’s mood states (Balas et al., 2012; Bolte et al., 2003), research has investigated further cognitive-affective processes underlying intuition and semantic coherence detection. The fluency-affect model of intuition suggests that processing fluency and subtle positive affect are major factors jointly driving coherence judgments (Topolinski & Strack, 2009a). Coherent triads are processed more fluently (i.e., faster), and fluency leads to a brief positive affective response channeling the intuitive judgment (e.g., a positive feeling of ease that is used in the following judgment or decision; Reber, Schwarz, & Winkielman, 2004; Reber, Winkielman, & Schwarz, 1998). Moreover, it has been shown that coherent triads are liked more than incoherent triads and that the mere reading of coherent triads activates people’s smiling muscle and relaxes the frowning muscle (indicating decreased negative affect and mental effort; see Topolinski, Likowski, Weyers, & Strack, 2009). These results suggest that coherence is fluently processed and triggers subtle positive affect that in turn functions as an internal cue generating the intuitive coherence judgment (Topolinski & Strack, 2009a; see also Winkielman & Cacioppo, 2001, for psychophysiological evidence on the effects of processing fluency and positive affect).

In addition, there is also evidence showing that manipulating both fluency and positive affect influences whether people feel coherence. Manipulating positivity on a subtle level (e.g., by subliminal affective facial priming or by manipulating the affective valence of word triads or solution words; Balas et al., 2012) increases participants’ tendency to judge triads as being coherent (independent of their actual coherence). In a similar vein manipulating the fluency of word triads (e.g., by manipulating the figure-ground contrast in which triads are presented) makes it more likely that people judge these as being coherent (as compared to less fluently processed word triads presented in a low figure-ground contrast; Topolinski & Strack, 2009a). Yet, whether manipulated fluency and positivity also influence depressed patients’ intuitive coherence judgments has not been investigated so far.

## The Current Study

The aim of the current study was to replicate and extend preliminary evidence on intuition deficits in depression. We tested a sample of depressed inpatients and compared their performance in the Judgment of Semantic Coherence Task (JSCT; Bowers et al., 1990) to a healthy control sample. Going one step further, we also aimed at investigating potential underlying mechanisms of impairments in intuitive coherence detection in patients with depression.

The following main hypotheses were pre-registered and investigated (see the Supplementary Materials for the preregistration): The first hypothesis (H1) refers to the replicability of recently found intuition deficits in depression (Remmers et al., 2015; Remmers et al., 2017). We hypothesized that patients with an acute episode of major depression are less able to intuitively discriminate semantic coherence from semantic incoherence in the JSCT than healthy controls.

The second hypothesis (H2a) assumes that processing fluency triggers semantic coherence judgments in patients with depression. Building up on basic research, we expected that in depressed patients word triads that are presented in a high figure-ground contrast – and which are therefore presumed to be processed more fluently – are more likely to be judged to be coherent than triads presented in a low contrast. Given that research using self-reports (Tsourtos et al., 2002; see also O’Connor et al., 1990) as well as experimental tasks supports the notion that mental activity is slowed in depression (e.g., Den Hartog et al., 2003), we also expected that the effect of processing fluency on coherence judgments would be smaller in the depressed sample as compared to the healthy sample (H2b).

The third hypothesis (H3a) was that the positive valence of stimuli influences semantic coherence judgments in patients with depression. Building up on basic research (Topolinski & Strack, 2009a; Experiment 8) showing that healthy subjects are more likely to judge triads to be coherent that consist of positive as compared to negative words, we expected that this effect would also emerge for depressed patients. However, it seemed reasonable to assume that depression moderates the effect of positive valence on coherence judgments because research shows that the preference for positive stimuli usually found in healthy samples is attenuated in depressed patients (Deveney & Deldin, 2006; Joormann & Gotlib, 2007). Thus, we hypothesized that the effect of positive valence on coherence judgments is smaller in the depressed sample as compared to the healthy sample (H3b). An a priori power analysis can be found in Appendix A.

## Method

### Participants

Forty inpatients were recruited from the Vivantes Klinikum Berlin-Kaulsdorf, Germany, a municipal psychiatry. The clinic staff informed the trained research assistant from the Freie Universität Berlin about patients potentially fitting the inclusion criteria, who were then approached in person. In addition, patients were addressed in the weekly psychoeducation depression group therapy. The healthy control sample was recruited through advertisements in social media, local newspapers and online advertisement platforms and tested by research assistants at the Freie Universität Berlin. In the clinical sample, the presence of a current episode of unipolar depression was required for inclusion. Exclusion criteria for the clinical sample were presence of psychotic symptoms, a bipolar disorder and acute suicidal tendencies. For the control sample, the presence of any mental disorder was an exclusion criterion. For all participants inclusion in the study additionally required a minimum age of 18 years and signed written consent. The inclusion and exclusion criteria were verified by conducting the affective and psychotic disorder modules of the Structured Clinical Interview according to DSM-IV with each participant (SCID; German version: Wittchen, Zaudig, & Fydrich, 1997). In the clinical sample, five subjects were excluded from the study. Two subjects did not fulfill the criteria for a current depressive episode. One subject had to be excluded due to the presence of psychotic symptoms and in one patient a depressive diagnosis due to a medical condition could not be excluded. Another subject could not credibly distance herself from suicidal tendencies during the interview, so that the hospital staff was called in and participation in the study was terminated. In the healthy sample, no subject was excluded. A total of 70 subjects took part in the study (35 in each group).

### Procedure

Upon arrival both at the clinic and at the University laboratory, participants were welcomed and received the informed consent that they were asked to sign. Participants were then interviewed by a trained rater with the SCID. Either directly after the SCID interview or at an appointment shortly afterwards, included subjects completed the intuition task consisting of three blocks (general intuition, fluency, valence; for a detailed description see Appendix B).

The procedure of the JSCT was identical to that of previous studies (Remmers et al., 2015, 2017). Participants saw a set of word triads (e.g. DEEP SALT FOAM; DREAM BALL BOOK) and were asked to indicate for each triad whether it was coherent or incoherent by pressing the respective key on the computer keyboard. Each trial began with the presentation of a fixation cross (1000 ms), followed by the presentation of the triad (1500 ms). After disappearance of the triad from the screen, "coherent" and "incoherent" appeared on the left or right side of the computer screen. The key positions of "coherent" and "incoherent" were randomized for each participant; once assigned, the key positions remained the same for each participant throughout the experimental task. Participants had 2000 ms to press the reaction key on the keyboard for their corresponding coherence judgment. If a participant failed to react within 2000 ms, "too slow" appeared on the screen and the next trial started. If participants managed to respond within the given reaction time window, they could type in an X or a possible solution word within 8 seconds. Each word triad was only presented once, which prevented exposure and repetition effects as well as analytic insight.

All participants performed three blocks that followed the procedure above but with varying stimulus material (see Appendix B for a detailed description). In the general intuition block, only coherence (coherent triads vs. incoherent triads) was manipulated. In addition to manipulating coherence, we manipulated fluency (high figure-ground contrast vs. low figure-ground contrast) in the fluency block and valence (positive triads vs. negative triads) in the valence block, resulting in four conditions in the latter two blocks respectively. At the end of each block participants indicated how much they trusted their intuition in the respective task on a 7-point Likert scale. All three blocks were programmed using jsPsych, a JavaScript library for creating behavioral experiments in a web browser (de Leeuw, 2015).

After completion of the three intuition blocks, subjects filled out a demographic questionnaire as well as other self-report instruments, not of interest for the current paper (see Supplementary Materials for all measured variables) and then received an AMAZON voucher as study compensation. Participating in the entire study lasted about 1 – 1.5 hours. The study was approved by the ethical committee of the Freie Universität Berlin and in compliance with the Helsinki Declaration.

### Statistical Analysis

Participants’ performance in the JSCT was the main outcome of the study. Trials were discarded in which participants did not provide their coherence judgment within the given time window of 2000 ms. These missed trials were analyzed separately and served us to explore whether patients and healthy controls differed in their ability to react within the given short time window. Solved trials were also discarded from the following coherence judgment analyses because these trials were indicative of explicit insight and not intuition (see Topolinski & Strack, 2009a). A trial was considered as solved when a participant provided the correct solution word or a synonym after the coherence judgment, being rated by two raters independently. Solved trials were thus analyzed separately and served us to explore whether depressed patients and healthy controls differed in the extent to which they had explicit insight. Participants who had missed responding in the given time window were not asked to type in a solution word. Missed trials and solved trials did not overlap.

To test H1, we computed a discrimination index for each participant after exclusion of missed responses and solved trials. For this, we first computed hit rates (i.e., the proportion of coherent trials that were correctly judged as coherent, but which were not solved) and false alarm rates (i.e., the proportion of incoherent trials, which had incorrectly been judge as coherent). We then calculated a simple discrimination index by subtracting false alarm rates from hit rates (called P_r_ in Snodgrass & Corwin, 1988; see also Bolte & Goschke, 2008). This index conveys participants’ ability to discriminate between coherent and incoherent trials (see Bolte et al., 2003; Remmers et al., 2017; Topolinski & Strack, 2009a). Participants’ responses are defined as accurate to the extent that their hit rate exceeds their false alarm rate. We tested H1 using an independent samples *t*-test with depression as the independent variable and the discrimination index from the JSCT general intuition block as the dependent variable.

Hypothesis H2a was tested using a random intercept model, which is conceptually equivalent to fitting a repeated measures ANOVA. In this model, the four conditions of the JSCT fluency block are nested within participants (i.e., each participant contributes four data points, and the random intercept accounts for the fact that the four assessments are usually positively correlated). We used coherence, fluency, and their interaction to predict the percentage of triads that have been judged as coherent (after deleting missed and solved triads). The relevant effect here was the effect of fluency. Note that we only included participants with depression for testing this hypothesis. In contrast, H2b was tested in the full sample, again using a random intercept model. This time, we used coherence, fluency, depression, and their two- and three-way interactions to predict the percentage of triads that have been judged as coherent in the fluency block. The relevant effect here is the interaction effect of depression and fluency. Hypotheses 3a and 3b were tested using the same approach, this time based on data from the JSCT valence block and using valence instead of fluency as a predictor.

In line with the preregistration, we corrected univariate outliers within groups (|z| > 2.5) prior to hypothesis testing using the winsoring method. This way, we corrected 19 data points in 21 variables across 70 participants (1.3%). The criterion for inferences for each hypothesis was *p* < .05 (two-tailed). Satterthwaite’s approximations were used to derive *p*-values for fixed effects in random intercept models. All analyses were conducted within the statistical environment R (R Core Team, 2018). Random intercept models were estimated using full maximum likelihood estimation implemented in the R package “lme4”.

## Results

### Descriptive Statistics

The depressive sample (*M* = 41.74, *SD* = 12.40) and the control sample (*M* = 44.37, *SD* = 16.85) did not differ significantly from each other in terms of age, *t*(62) = 0.74, *p* = .46. Also, with respect to gender (depressed group: 22 females; control group: 21 females), there was no significant group difference, χ^2^(1) = 0.06, *p* = .806. However, there was a significant difference in terms of education, *U*(35, 35) = 445.5, *p* = .032, with the control sample having a higher educational degree as compared to the depressed sample.

### Preparatory Analyses of the General Intuition Block

Results suggested that depressed patients did not differ significantly from healthy controls regarding the number of missed trials (i.e., trials in which subjects did not respond within the given time window), the number of solved trials (i.e., coherent trials for which the correct solution word was typed in), and the average response time (see Appendix C for details). Moreover, depressed patients (*M* = 0.50, *SD* = 0.18) and healthy control participants (*M* = 0.47, *SD* = 0.21) did not differ significantly in the hit rate (i.e., proportion of triads that they correctly judged as coherent), *t*(68) = 0.68, *p* = .50, 95% CI [-0.06, 0.13]. Also, with regard to the false alarm rate (i.e., proportion of triads that were incorrectly classified as coherent), there was no significant difference between the depressed sample (*M* = 0.32, *SD* = 0.17) and the healthy sample (*M* = 0.28, *SD* = 0.14), *t*(68) = 1.01, *p* = .31, 95% CI [-0.04, 0.11]. Finally, on average, participants from both samples could discriminate between coherent and incoherent trials above chance level. This was indicated by one sample *t*-tests showing that the discrimination index differed from zero in both the depressed sample (*M* = 0.18, *SD* = 0.17, *t*[34] = 6.13, *p* < .001) and the healthy sample (*M* = 0.19, *SD* = 0.18, *t*[34] = 6.10, *p* < .001).

### Confirmatory Hypotheses Testing

**Figure 1:**
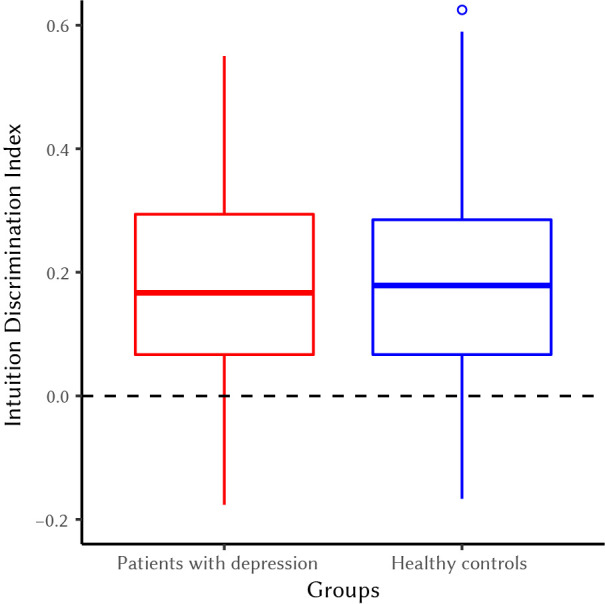
Boxplots of Intuition Discrimination Index for Patients With Depression (Red Column) and Healthy Control Participants (Blue Column)

Analyses regarding H1 revealed that depressed patients and healthy control participants did not differ significantly in their ability to discriminate semantic coherence from semantic incoherence in the JSCT general intuition block, *t*(68) = - 0.12, *p* = .90, 95% CI [-0.09, 0.08] (see Figure 1).

**Figure 2:**
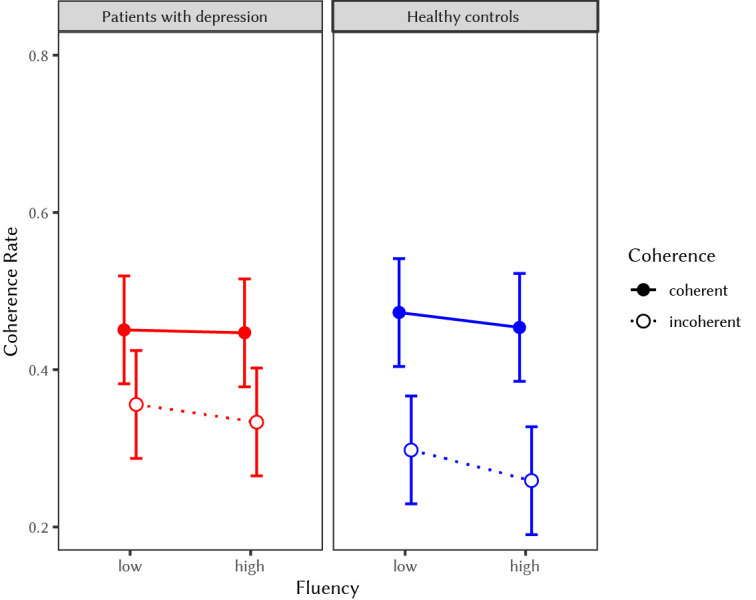
Coherence Judgment Rates for Depressed Patients and Healthy Controls in the High vs. Low Fluency and Coherent vs. Incoherent Conditions *Note.* Figure 2 indicates no significant effect of the fluency manipulation on coherence judgments and no interaction effect between group and fluency on coherence rate.

With regard to H2a, results showed that fluency did not significantly predict the percentage of triads that have been judged as coherent in depressed patients, *F*(1,105) = 0.21, *p* = .65. However, coherence had a significant effect on coherence judgments, with coherent trials being more likely to be judged as coherent as compared to incoherent trials, *F*(1,105) = 13.57, *p* < .001. The interaction between coherence and fluency was not significant, *F*(1,105) = 0.11, *p* = .74 (see left panel in Figure 2).

With regard to H2b, results revealed that the interaction effect of depression and fluency was not significant in predicting coherence judgments, *F*(1, 210) = 0.19, *p* = .66. Thus, our findings do not support the hypothesis that the effect of fluency on coherence judgments was smaller in the depressed sample as compared to the healthy sample. In this model, only coherence, *F*(1, 210) = 60.95, *p* < .001, and the interaction of group and coherence, *F*(1, 210) = 4.75, *p* = .03, significantly predicted the percentage of triads that have been judged as coherent. Fluency, depression, and further interaction effects were not significant (see Figure 2).

**Figure 3:**
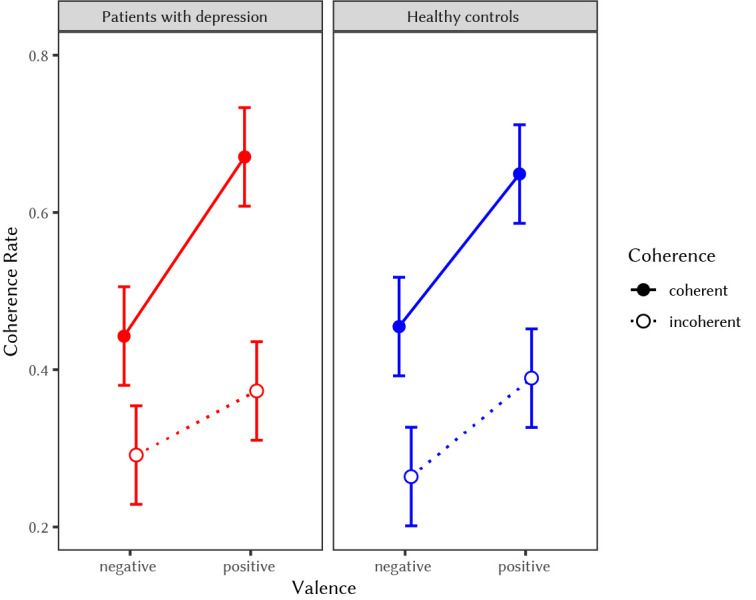
Coherence Judgment Rates for Depressed Patients and Healthy Controls in the Positive vs. Negative Valence and Coherent vs. Incoherent Conditions *Note.* Figure 3 indicates a significant effect of the valence manipulation in both the depressed group and the control group and no interaction effect between group and valence on coherence rate.

In line with H3a, analyses revealed that positive valence of word triads significantly predicted semantic coherence judgments in depressed patients, *F*(1, 105) = 38.45, *p* < .001. Furthermore, the effect of coherence, *F*(1, 105) = 80.98, *p* < .001, and the interaction effect of valence and coherence were significant, *F*(1, 105) = 8.60, *p* < .01. The pattern of results suggested that coherent triads that were positive in valence were most likely to be judged as coherent (see left panel of Figure 3).

With respect to H3b, results did not confirm the hypothesis that depression moderated the effect of positive valence on coherence judgments. The effect of positive valence on coherence judgments was not smaller in the depressed sample as compared to the healthy sample, *F*(1, 210) = 0.02, *p* = .88. In this model, valence, *F*(1, 210) = 85.52, *p* < .001, coherence, *F*(1, 210) = 175.04, *p* < .001, and their interaction, *F*(1, 210) = 10.01, *p* < .01, were significant. The effect of group and its interaction with valence and coherence were not significant (see Figure 3).

### Exploratory Follow-up Analyses

For testing our main preregistered hypothesis regarding intuition deficits in depressed patients, we selected a simple discrimination index (i.e., the difference between hit and false alarm rates) that has also been used in previous studies (Bolte & Goschke, 2008). To check the robustness of our results, we calculated another index established in signal detection theory, namely A’. This non-parametric measure accounts for small numbers of observations per cell and corrects for hit rates of 1.0 and false-alarm rates of 0.0 (Pollack, 1970; Pollack & Norman, 1964). A’ of .5 indicates performance on chance level, and perfect discrimination yields an A’ of 1. We repeated our analyses for H1 using A’ and found that results did not differ from results using the simple discrimination index (see Appendix C).

We also repeated our preparatory analyses and analyses for H1 combining the data from all blocks (i.e., including the data from the fluency and valence block). With regard to H1, again no significant differences emerged, neither for the simple discrimination index nor for A’. We also did not find significant differences in the number of solved trials. However, across all intuition blocks, depressed patients missed more trials and had significantly higher reaction times as compared to healthy controls (see Appendix C).

## Discussion

The aim of the present study was to replicate recently demonstrated deficits in intuitive semantic coherence detection and to explore the effects of processing fluency and positivity on intuition in depressed patients. One major finding was that patients with depression did not differ from healthy controls in their ability to discriminate semantic coherence from semantic incoherence (H1). Even though controls were better at discriminating coherent from incoherent triads compared to depressed patients in the fluency block (indicated by a significant interaction between group and coherence), differences in discrimination indices were not significant when considering data from all blocks.

These findings may query the hypothesis of impaired intuition in depression. However, methodological issues should be considered. The true difference between the groups might be smaller than expected (based on prior research). Thus, our study may have lacked the power to detect it. Moreover, hit and false alarm rates were computed after exclusion of missed trials. Thus, subjects who only responded when they were relatively confident in their judgment (i.e., when seeing a comparably easy triad) might have yielded a higher intuitive discrimination index as compared to subjects who missed relatively few trials. As such, the non-significant difference in intuitive performance might have resulted from depressed patients’ higher tendency to not respond within the given time window, for example when being unsure and less confident, or when confronted with more difficult trials. Indeed, depressed patients missed significantly more trials as compared to healthy control participants when considering their responses across all three intuition blocks.

Our exploratory analyses with reaction times also showed that on average and across all blocks, depressed patients were slower than healthy controls. Together with the finding on missed trials, this result points out that future research would do well in elucidating how longer response times are associated with patients’ intuitive discrimination accuracy. Researchers should hereby distinguish between simple between-subject approaches such as mean reaction time analyses and more sophisticated within-subject methodologies. Using, for example, stochastic diffusion models, can provide important insights into speed-accuracy trade-offs (Voss, Nagler, & Lerche, 2013). The latter take information from individual distributions into account and hereby help to disentangle how performance differs between conditions (or groups), and – importantly – in which way it does and how speed-accuracy interactions may reflect cognitive biases (e.g., being more accurate when responding faster may reflect intuitive capacities). Although this kind of model can be applied to many experimental paradigms and provides much more insight than the analysis of mean response times, it is still rarely used in cognitive psychology and in clinical psychopathology research in specific.

Altogether, our findings and considerations call for more replication studies to elucidate the question whether depressed patients are impaired in intuitively detecting meaning and coherence in their environment and within themselves (e.g., meaning in life). Regarding the latter, it seems fruitful to connect intuitive coherence detection research with research on memory coherence, i.e., the ability to construct one’s autobiography in a coherent, integrated way. As memory coherence is associated with psychological health, positive therapy outcomes and seems to buffer protectively against the impact of early life stress (Adler et al., 2013; Baerger & McAdams, 1999), future research should explore to what extent performance in the JSCT is associated with a person’s memory coherence. Hereby, upcoming research should also take the heterogeneity of depression (Monroe & Anderson, 2015) as well as interindividual differences into account. Hicks and colleagues (2010) showed for example how interindividual differences in self-reported preference for intuitive processing influence the interplay between positive affect and intuition. With respect to our research question arises whether intuitive processes are especially impaired in patients with recurrent forms of depression, (and) or only in patients with anhedonia? In other words: It should be explored for which patients the assumption of impaired intuitive processing holds to get a better understanding of this issue.

Results did not reveal any effect of our fluency manipulation, and thus our hypotheses regarding fluency (H2) were not supported. As such, the current study could not replicate previous results that bolstered the fluency model proposed by Topolinski and Strack (2009a, 2009b). In order to explore whether processing fluency will prove as a major determinant of coherence judgments or not, future studies should use other fluency manipulations such as priming (see Topolinski & Strack, 2009a). In addition, future research should take into account that fluency may not always lead to positive affective responses (Gamblin, Banks, & Dean, 2020) and thus also not to coherent responses. Given that processing fluency and affective responses may interact differently depending on characteristics of the presented stimulus or the responding individual, future studies should disentangle the differential effects of processing ease on task performance.

The results further showed that positive (vs. negative) valence triggered coherence judgments (H3a) and that this effect was – in contrast to our hypothesis (H3b) – not moderated by depression. This suggests that depressed patients may be susceptible for positive affectivity conveyed by the valence of word triads and used it – when provided externally – in their judgments. This is an important finding, because even though depression is characterized by anhedonia (i.e., the inability to experience positivity), patients seemed to be inclined to detect meaningfulness and coherence when encountering positive valence, bolstering the idea that positivity plays a major role in finding meaning.

How can the current findings be reconciled with previous research depressed patients’ processing of positivity? At first glance they seem to stand in contrast to research showing that – opposed to healthy people – depressed patients do not direct their attention to positivity and are less susceptible to positive stimuli (Duque & Vázquez, 2015; Pool, Brosch, Delplanque, & Sander, 2016; Winer & Salem, 2016). In their comprehensive review LeMoult and Gotlib (2019) conclude that biases (e.g., faster reaction time in response to negative as opposed positive stimuli) are mostly found when stimuli are presented longer and when faces as opposed to words are presented. Thus, it is conceivable that depression did not moderate the effect of positive valence manipulation in our study because (a) presentation of stimuli was short enough (and hereby prevented conscious processing) and (b) words (and no faces) were presented. However, these comparative conclusions should be drawn cautiously because our main outcome were binary coherence judgments and not response times. Given the heterogeneity of previous research on biases in the processing of positive affect (e.g., Yoon, Joormann, & Gotlib, 2009), future studies would do well in examining different cognitive abilities (memory, attention, intuitive decisions) along together. Also, it remains open to what extent positivity exhibited its effect on a conscious level. Future work should elucidate this issue by exploring whether rather implicit or explicit induced positive affect resonates in depressed patients.

Our findings also revealed a significant interaction between valence and coherence in the valence block (i.e., positive word triads that were coherent were most likely to be judged as coherent in both groups). This finding indicates that positivity (conveyed by positive valence in the current study) may lead to more accurate intuitive judgments and is in line with previous research showing that not only “tonic” positive affect (e.g., manipulated or freestanding positive mood; Balas et al., 2012; Bolte et al., 2003) but also “phasic” positive affect (induced by the activation of positively valenced memory content) can strengthen the accuracy of coherence judgments (Topolinski & Strack, 2009b). In a similar fashion, Balas et al. (2012) demonstrated increased accuracy for triads with positive solution words as compared to triads with negative solution words. Future research should test the underlying theoretical assumptions on the positive affect-intuition-interplay by implementing measures assessing positive affect in individuals, because otherwise it remains speculative whether it is indeed “affect” (within the individuals) that triggers these effects (see Alves et al., 2015, for potential alternative explanations on the effects of positive valence).

Along this line, it is of important practical relevance to test whether depressed patients can themselves produce the positive affect needed to go with their intuition in daily life. Extending laboratory research, a recent daily diary study found that people are not only inclined to make decisions intuitively when they are in a good mood (as compared to a negative mood, Remmers & Zander, 2018) but that people also report to feel better after intuitive as compared to analytical decisions (Zander-Schellenberg, Remmers, Zimmermann, Thommen, & Lieb, 2019). To explore whereas these decision-mood dynamics also apply to currently depressed patients outside the laboratory, is an important next step also in terms of ecological validity and clinical relevance.

From a therapeutic perspective, the current findings imply that targeting positive affect in psychotherapy may be important in fostering patients’ ability to detect coherence. It would be an important next step to investigate the intuitive detection of meaning not only with regard to laboratory stimuli but also on a broader level with regard to finding coherence and meaning in life. Hereby, clinical researchers may build upon recent basic psychological research on how intuitive processing, positive affect, and finding meaning in life interact (Heintzelman & King, 2013). Considering that finding meaning in life is rather a product of intuitive processing than a result of analytical reasoning or explicit meaning construction (Heintzelman & King, 2013), research in this field may have far-reaching practical and theoretical clinical implications.

A number of limitations should be taken into account. First, even though the sample size was in compliance with the a-priori power analysis, it was still relatively small. Thus, future studies should test our assumptions with larger samples to increase the power and reliability of findings. Furthermore, a limitation of the current study was that the samples were not matched in terms of educational level. Even though the relatively lower educational level of depressed patients is consistent with epidemiological studies showing that the prevalence of psychological disorders is higher in low socioeconomic groups, future studies should take care of the matching issue to avoid potential confounds. In addition, we randomized different factors such as the key position for the coherence judgments and the stimuli that were either presented in the main intuition block or the fluency bock. Even though randomization is of methodological importance, this may have led to reduced comparability of responses between subjects and thus to reduced power to detect group differences. Thus, future research should use larger sets of stimuli and larger sample sizes in order to ensure randomization and reduce statistical noise. Furthermore, conclusions with regard to the role of positivity should be drawn cautiously because our study was lacking a neutral control condition. Thus, we cannot rule out that, for example, reduced negativity (as opposed to increased positivity) drove our effects in the valence block. Future studies should test whether positivity (e.g., conveyed by the valence of stimuli) alters subtle affective responses in subjects. Only by this means we can conclude whether positive affect elicited in subjects triggers coherence judgments (see Topolinski & Strack, 2009a for a detailed description of the fluency-affect intuition chain).

Albeit these considerations, the current study presents an important contribution to the field. It is a preregistered replication study which follows current state-of-the-art demands to bolster the robustness of psychological research findings. In addition, we used experimental paradigms from basic psychology and hereby build the bridge from basic to clinical research. Altogether one may conclude from the current study that the cognitive profile of depressed patients is not merely deficient. The results elucidate the importance of positivity when it comes to detecting meaning and coherence. The latter is of major clinical importance, because in a depressed state, people often experience their life as meaningless and cannot find coherence. Whether promoting positivity may not only enhance how patients feel but will also help them to find meaningfulness and to follow their intuitions is a fruitful endeavor to study for future research.

## Supplementary Materials

The supplementary materials include the preregistration protocol for this study (for access see Index of Supplementary Materials below).



RemmersC.
ZimmermannJ.
TopolinskiS.
RichterC.
Zander-SchellenbergT.
WeilerM.
KnaevelsrudC.
 (2020). Supplementary materials to "Intuitive judgments in depression and the role of processing fluency and positive valence: A preregistered replication study"
[Preregistration protocol]. PsychOpen. https://osf.io/5fpwk10.32872/cpe.v2i4.2593PMC964547036398058

## Appendix A: Power Analysis

Where possible, we based our power analyses on the effect sizes found in earlier work. In particular, the effect size of the intuition impairment in patients with depression compared to healthy controls was *d* = 0.71 (H1; Remmers et al., 2015); the within-subjects effect size of the fluency manipulation in a student sample was *d_z_* = 0.5 (similar to H2a; Topolinski & Strack, 2009a, Experiment 1); and the within-subjects effect size of the affect induction in a student sample was *d_z_* = 1.19 (similar to H3a; Topolinski & Strack, 2009a, Experiment 8). We did not have any prior information about the size of the proposed interaction effects (H2b and H3b), and thus we considered here a smaller effect size of *d* = 0.35. Based on these assumptions, we conducted a series of a priori power analyses using GPower (Faul, Erdfelder, Buchner, & Lang, 2009), focusing on the difference between two independent means (H1), the within-subjects effect of a repeated measures ANOVA (H2a and H3a, assuming a correlation of *r* = .5 between the repeated measurements), and the within-between-subjects interaction effect of a repeated measures ANOVA (H2b and H3b, again assuming a correlation of *r* = .5 between the repeated measurements). To detect each of these effects with a probability of 80% and an alpha error probability of 5% (two-sided), the following sample sizes (per group) are required: 33 (H1), 34 (H2a) 34 (H2b), 8 (H3a), and 34 (H3b). Thus, we conclude that 35 participants per group may represent an acceptable sample size given prior findings.

## Appendix B: Procedure of the Judgment of the Semantic Coherence Task

**Introduction Phase**. The first computer screens introduced subjects to the intuition task and explained that the task was about intuition. Participants were informed that the task was not about right or wrong decisions or about finding a solution (i.e., typing in the correct solution word) but rather about intuitive gut reactions in response to the presented stimuli. Letting participants type in a solution word served us to distinguish between intuitively detected but unsolved trials (being indicative for intuition; see Remmers et al., 2015 and Topolinski & Strack, 2009a for detailed description) and explicitly solved trials (being indicative for insight and not intuition). The introductory phase also included the presentation of exemplary coherent and incoherent word triads (e.g., DEEP SALT FOAM; coherent triad, common denominator: SEA) not reappearing later in the task. Next, subjects underwent a practice block in which they were asked to react within 2000 ms and to indicate whether presented exclamation marks appeared on the right or left side of the screen by pressing the respective keyboard keys. The same keys, namely S and L on the keyboard (German QWERTZ keyboard layout), later served as reaction keys for the coherence judgments. Keyboard button assignment was randomly assigned for each participant and remained the same for each participant once assigned during the entire experimental task. Hereby, it was manipulated whether the left (S) or the right (L) keyboard button indicated a coherence or incoherence judgment.

**General Intuition Block**. To measure the general ability to intuitively detect semantic coherence subjects performed the JSCT according to Bowers et al., 1990 (see also Topolinski & Strack, 2009a; Remmers et al., 2015, 2017). Thus, in this block, all subjects performed the JSCT in which only the coherence of the triads was manipulated (coherent vs. incoherent word triads). A total of 36 triads were presented in this part to test the ability to detect semantic coherence (18 coherent, 18 incoherent, re-randomized order for each subject, stimulus material see Bolte et al. 2003; Topolinski & Strack, 2009a).

**Fluency Block**. To investigate the effect of processing fluency on coherence judgments, an experimental manipulation established in basic research was applied. Methodologically equivalent to Topolinski and Strack (2009a, Experiment, 1), the figure-ground contrast was manipulated as a means to alter the fluency with which stimuli are processed. For this, triads were presented in blue, red or green letters. High-fluency triads had a high figure-ground contrast (against the white background) by manipulation of the RGB (red, green, blue) components. An RGB combination of R = 255, G = 0 and B = 0 results, for example, in a red triad with a strong contrast, whereas the combination of R = 255, G = 200, B = 200 yields a light red colour and hence low contrast against the white background. In line with the procedure of Topolinski & Strack (2009a; but see also Reber, Winkielman, & Schwarz, 1998; Unkelbach, 2007), we designed a red high-contrast (thus high-fluency) triad by assigning a random value between 100 and 120 for the B and G component, and by assigning 255 to the R component. A red low-contrast (thus low-fluency) triad was designed by assigning a random value between 200 and 220 for the B and G components. This was one for the other colours, too. Deriving from the stimulus pool of Bolte et al. (2003), 36 triads were presented. Using a 2 (high vs. low fluency) x 2 (coherent vs. incoherent) intra-individual factorial design, 4 experimental conditions resulted: 9 coherent triads with high figure-ground contrast; 9 incoherent triads with high figure-ground contrast; 9 coherent triads with low figure-ground contrast; 9 incoherent triads with low figure-ground contrast. It should be noted that stimuli of the general intuition block and the fluency block are taken from the same stimulus pool, but are randomly selected anew for each participant ensuring that no triad is seen twice by an individual on the one hand and that whether a triad is presented in the intuition or the fluency block is a matter of randomization and not preselected by the study team.

**Valence Block**. To investigate the effect of positive valence on coherence judgments, subjects are presented with positive (e.g., LUCK CHILDREN MEADOW; common denominator: GAME) and negative (e.g., SULFUR GLUE BLACK; common denominator: PITCH) word triads. Just like in the other two blocks, subjects’ task is to decide whether a presented triad is coherent or incoherent. Taken from the stimulus pool of Topolinski and Strack (2009a; Experiment 8), 48 triads were presented to each subject. Using a 2 (positive vs. negative) x 2 (coherent vs. incoherent) intra-individual factorial design, 4 experimental conditions resulted: 12 positive coherent triads; 12 positive incoherent triads, 12 negative coherent triads, 12 negative incoherent triads).

## Appendix C: Preparatory Analyses and Exploratory Follow-up Analyses

**Table C.1:** Preparatory Analyses and Exploratory Follow-up Analyses

Variable	Patients (*n* = 35)		Controls (*n* = 35)		*t*-tests						
	*M*	*SD*	*M*	*SD*	*ΔM*	*t*	*df*	*p*	*CI_low*	*CI_high*	*d*
General Intuition Block											
Discrimination Index	0.18	0.17	0.19	0.18	0.00	-0.12	68	0.90	-0.09	0.08	-0.03
A‘	0.65	0.13	0.65	0.14	0.00	-0.02	68	0.98	-0.06	0.06	-0.01
Number of missed trials	7.17	4.73	5.17	4.23	-2.00	1.86	68	0.07	-0.14	4.14	0.45
Number of solved trials	0.63	0.91	0.89	1.05	0.26	-1.09	68	0.28	-0.73	0.21	-0.26
Average reaction time per trial (in seconds)	1.11	0.15	1.04	0.20	-0.08	1.79	68	0.08	-0.01	0.16	0.43
Combined blocks											
Discrimination Index	0.17	0.11	0.20	0.10	0.03	-0.98	68	0.33	-0.08	0.03	-0.24
A‘	0.64	0.09	0.66	0.09	0.02	-1.07	68	0.29	-0.06	0.02	-0.26
Number of missed trials	15.90	10.76	10.85	8.79	-5.05	2.15	68	0.04	0.36	9.74	0.52
Number of solved trials	1.87	2.47	2.19	2.26	0.31	-0.55	68	0.58	-1.45	0.82	-0.13
Average reaction time per trial (in seconds)	1.02	0.20	0.93	0.19	-0.10	2.11	68	0.04	0.01	0.19	0.51
